# Feasibility and Efficacy of Morning Light Therapy for Adults with Insomnia: A Pilot, Randomized, Open-Label, Two-Arm Study

**DOI:** 10.3390/medicina59061066

**Published:** 2023-06-01

**Authors:** Jihyun Yoon, Seokjae Heo, Hyangkyu Lee, Eungyeong Sul, Taehwa Han, Yu-Jin Kwon

**Affiliations:** 1 Department of Family Medicine, Korean University Anam Hospital, Seoul 02481, Republic of Korea; ghyun.yoon@gmail.com; 2 Division of Biostatistics, Department of Biomedical Systems Informatics, Yonsei University College of Medicine, Seoul 03722, Republic of Korea; sjheo@yuhs.ac; 3 College of Nursing, Mo-Im Kim Research Institute, Yonsei University, Seoul 03722, Republic of Korea; hkyulee@yuhs.ac; 4 Department of Family Medicine, Yonsei University College of Medicine, Seoul 03722, Republic of Korea; lovelyjhy@hanmail.net; 5 Health-IT Center, Yonsei University Severance Hospital, Seoul 03722, Republic of Korea

**Keywords:** light therapy, sleep disorder, daytime sleepiness, circadian rhythm, clock genes

## Abstract

*Background and Objectives:* Light therapy (LT) is used as an adjunctive treatment for sleep problems. This study evaluates the impact of LT on sleep quality and sleep-related parameters in patients with sleep disorders. *Materials and Methods:* We performed a pilot, randomized, open-label clinical trial. Fourteen patients aged 20–60 years with insomnia were randomized into the control and LT groups (1:1 ratio). The LT group was instructed to use a device that provides bright LT (6000 K, 380 lux, wavelength 480 nm) for at least 25 min before 09:00 a.m. for two weeks. A self-reported questionnaire was used to evaluate circadian preference, mood, and sleep-related parameters. We analyzed serum cortisol levels and clock genes’ expression. *Results:* The Epworth Sleepiness Scale (ESS), insomnia severity index(ISI), and Pittsburgh Sleep Quality index(PSQI) were significantly improved within the LT group only after the two-week period. When comparing the two groups, only the change in ESS was significant (mean difference, control: −0.14 vs. LT: −1.43, *p* = 0.021) after adjusting for the baseline characteristics. There were no significant differences in serum cortisol or clock genes’ expression. *Conclusions:* LT can improve daytime sleepiness in patients with sleep disorders; however, further well-designed studies are warranted to confirm its efficacy.

## 1. Introduction

The influence of sleep on health has been verified in many previous studies [1,2]. Most studies proved that sleep is essential for maintaining basic human functions, such as cognition, emotions, and metabolism [3]. Sleep problems have been emerging as social and public health issues because they can affect daily activity and function [4] and promote the development of metabolic diseases, such as hypertension [5,6], diabetes [7], and obesity [8,9]. In addition, they can increase the risk of cancer [10], mental health conditions [11,12], and psychiatric disorders [13]. According to epidemiological studies, the prevalence of insomnia in the general population ranges from 6% to 20% [14,15]. In a similar fashion, a study in Republic of Korea has also confirmed that the incidence rate of insomnia is 1 out of 5 adults [16].

Recent studies have focused on the implication of the circadian system, which has a significant impact on the sleep–wake cycle, fluctuations in hormones’ secretion, cardiovascular health, and glucose homeostasis, in sleep problems [17]. In fact, circadian rhythms are biological cycles that allow human beings to adjust their physiology and behavior according to changes in the external environment. These cycles are regulated by the suprachiasmatic nucleus (SCN) of the hypothalamus. The circadian rhythm is guided by various environmental signals, of which light is the most influential [18]. Dramatic shifts in life patterns induced by technological development and cultural changes have fundamentally altered our external environment, and people’s circadian rhythms have been affected as well. In this context, circadian disruption and dysregulation in adults are associated with sleep disorders [18,19]. Therefore, it is necessary to prevent disharmony in the circadian rhythm and maintain a balanced state by providing light with the appropriate timing, intensity, duration, and wavelength, similar to natural light; however, modern city lifestyles make this difficult. There are many studies that investigate the utility of sources of artificial lighting in the management of sleep problems [19,20,21]. Pharmacological agents, cognitive behavioral therapy, and psychosocial interventions have been traditionally used to treat sleep problems, but light therapy (LT) is receiving more attention due to the limitations that accompany the traditional therapies, such as drug side effects, cost, and limitations of time and space [22,23]. LT is natural, simple, relatively inexpensive, and useful as an adjuvant treatment for sleep disorders due to its few interactions with other treatment options. A systematic review and meta-analysis, which involved 22 studies and 685 participants, demonstrated the significant improvement of wake after sleep onset with LT in managing insomnia, supporting the overall effectiveness of LT in treating sleep problems [21], as highlighted by another meta-analysis of 53 studies involving 1154 participants [19]. However, only a few studies have examined the effects of LT on sleep-related parameters. Therefore, this study aimed to investigate the improvement in circadian rhythm through LT and evaluate the effect of the latter on sleep quality and sleep-related parameters.

## 2. Materials and Methods

### 2.1. Study Design and Patients

This study was a pilot, randomized, open-label, two-arm clinical trial that was conducted over a period of two weeks. The study protocol was approved by the Institutional Review Board of Yongin Severance Hospital (IRB No. 9-2022-0025) (date of approval: 30 March 2022) and registered at the Clinical Research Information Service (CRIS, KCT0007293) (date of registration: 17 May 2022). This study was conducted in compliance with the principles of the Declaration of Helsinki. Informed consent was obtained from all subjects. Patients were recruited from 18 April to 27 June 2022, at Yongin Severance Hospital (Yongin, Republic of Korea). We recruited individuals who sought medical care at a primary care clinic due to persistent sleep problems lasting more than three months, which caused significant distress or impairment in their daily functioning. We specifically focused on primary insomnia, a chronic sleep disorder characterized by difficulties in initiating or maintaining sleep, or experiencing non-restorative sleep, without any underlying medical, psychiatric, or environmental causes. The exclusion criteria were as follows: sleep disorders included in the DSM-5, except for primary insomnia disorder (e.g., hypersomnolence disorder, narcolepsy, obstructive sleep apnea hypopnea, central sleep apnea, sleep-related hypoventilation, etc.), sleep disorders related to other mental disorders, sleep disorders related to other medical conditions, history of psychiatric illness, and patients starting other treatments for insomnia within three months. Only individuals who were taking sleep medication without altering the type or dosage during the trial period were included in the study. We also excluded individuals who were shift workers or had traveled through more than two time zones within the past month.

### 2.2. Randomization and Study Protocol

**Figure 1:**
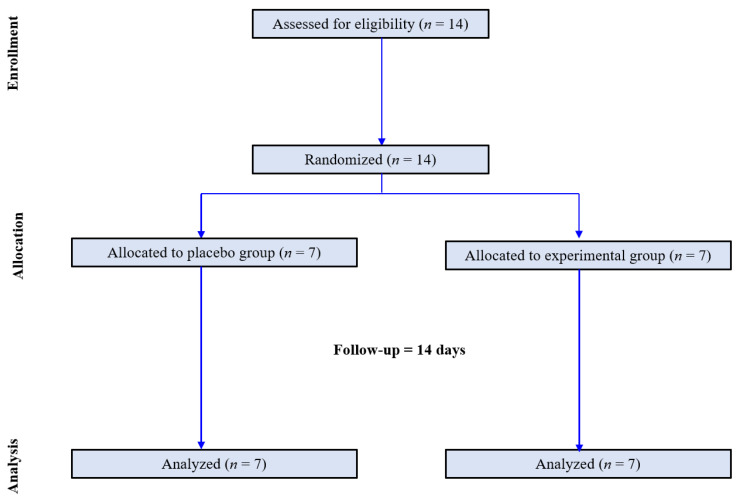
Flow chart of the study population.

Participants were randomly assigned in a 1:1 ratio to the control and LT groups. Randomization was performed using a centralized computer-generated system. During the two-week period, there were no dropouts. Finally, this study was completed by seven participants in the control group and seven in the LT group (Figure 1). The control group was instructed to maintain their usual daily life routines without any intervention. The LT group was provided with a small LT device (Olly, Samsung Electronics C-lab company, Luple, Seoul, Republic of Korea). The device emitted blue-enriched light (at a wavelength of 480 nm), designed to mimic the quality of morning sunlight. The light intensity was set at 380 lux, a level determined at eye level when the light box was placed 30 cm away from the participants’ faces. This intensity level was chosen based on previous studies showing that blue light at a similar intensity could elicit beneficial physiological changes in circadian rhythm regulation and overall sleep quality. Participants were instructed to maintain their gaze towards the light box for at least 25 min daily before 9:00 a.m. for two weeks. The timing for LT was set to align with the typical hours of morning sunlight exposure. This schedule was chosen because exposure to natural morning sunlight plays a crucial role in the regulation of our circadian rhythms. However, due to the constraints of this study, we were unable to ascertain the individual circadian rhythms of the participants. As such, the chosen timing of LT administration does not explain individual differences in circadian rhythms. Photometer assessment results by the Korea Photonics Technology Institute are presented in Supplementary Figure S1.

### 2.3. Clinical and Biochemistry Analyses

Study visits were scheduled at screening, baseline, and after two weeks. Body weight and height were measured. Body mass index (BMI) (kg/m^2^) was calculated as height in kilograms divided by the square of height in meters. Systolic blood pressure (SBP) and diastolic blood pressure (DBP) were measured in a sitting position using the right arm after at least 5 min of rest. Lifestyle (smoking, alcohol consumption, and exercise) and underlying diseases (diabetes, hypertension, and dyslipidemia) were assessed using a self-reported questionnaire. The participants were categorized into the following: current smoker or not and current drinker or not. Physical activity was defined as exercising for more than 30 min and more than 3 times a week. We used a binary variable for the presence or absence of a history of hypertension, dyslipidemia, or diabetes.

Although the exact indoor temperature and brightness of the hospital were not measured, it is estimated that the optimal indoor temperature within the hospital would have been maintained between 18 and 20 °C. All participants visited the hospital before 8 a.m. for their blood tests. Blood samples were obtained after more than 8 h of fasting. White blood cell (WBC) counts were quantified by flow cytometry using XN2000 (Sysmex, Kobe, Japan). Insulin levels were analyzed by the chemiluminescent microparticle immunoassay using the Architect i2000SR (Abbott, Abbott Park, IL, USA). Lipids (total cholesterol (TC), low-density lipoprotein cholesterol (LDL-C), non-high-density lipoprotein cholesterol (non-HDL-C), triglycerides (TG), and high-density lipoprotein cholesterol (HDL-C)) were analyzed using an enzymatic color test. C-reactive protein (CRP) levels were analyzed using an immunoturbidimetric method. The homeostasis model assessment of insulin resistance (HOMA-IR) was calculated using the following equation: fasting glucose (mmol/L) × fasting insulin (µU/mL)/22.5.

Cortisol and adrenocorticotropic hormone (ACTH) levels were assessed using an electrochemiluminescence immunoassay (Roche Cobas 8000 e801, Mannheim, Germany). Serotonin levels were assessed by liquid chromatography-MS/MS using an electrochemiluminescence immunoassay (SCIEX, 5500 Qtrap, Framingham, MA, USA).

A heart rate variability (HRV) analyzer with a three-lead electrocardiogram (Medicore Co., Ltd., Gyeonggi, Republic of Korea) was used for 5 min in the supine position in a quiet room. Common HRV indices, such as the standard deviation of normal-to-normal intervals (SDNN), total power (TP), low frequency (LF), high frequency (HF), and the LF/HF ratio, were measured.

### 2.4. Actigraphy

All participants wore a wrist actigraphy device (ActiGraph wGT3X-BT, ActiGraph, Pensacola, FL, USA). This actigraphy device was worn on the non-dominant wrist using a wrist strap. The participants were asked to wear the device continuously throughout the day. The device was set to sampling counts per one-minute epochs. Actigraphy data were analyzed using the ActiLife software (ActiGraph LLC, Pensacola, FL, USA). During the two weeks, sleep efficiency, total sleep time, time in bed, wake after sleep onset, and number of nocturnal awakenings were measured using actigraphy. To minimize heterogeneity, sleep habits were recorded daily using a sleep diary.

### 2.5. Sleep-Related Questionnaire

A self-report questionnaire was used to evaluate circadian preference, mood, and sleep-related parameters. Mental health metrics were assessed using the Patient Health Questionnaire (PHQ-9). The PHQ-9 assesses depression severity over the past two weeks. It is comprised of 9 items, and the total score is 27 points. A PHQ-9 score ≥ 10 is considered as major depression [24]. The Korean version of the Morningness–Eveningness Questionnaire (MEQ) was used to assess the circadian preference [25,26]. The MEQ consists of 19 questions that ask people to consider their “feeling best” rhythms and indicate their preferred sleep time and daily performance in various everyday life. The scores range from 16 to 86 points. A higher score indicates a morning preference. Overall sleep quality and sleep disturbance were measured using the Pittsburgh Sleep Quality Index (PSQI), one of the most widely used sleep diagnostic questionnaire tools [27]. The total PSQI score ranges from 0 to 21, with higher scores indicating poorer sleep quality. The insomnia severity index (ISI) is a self-report tool that measures subjective symptoms, consequences of insomnia, and the degree of concerns and distress [28]. The total score ranges from 0 to 28. A higher score indicates more severe insomnia. The Stanford Sleepiness Scale (SSS) is a self-rating scale that measures a patient’s subjective evaluation of sleepiness on a seven-point Likert-scale [29]. The Epworth Sleepiness Scale (ESS) consists of 8 question and ranges from 0 to 24. A higher score indicates a higher level of daytime sleepiness [30].

### 2.6. Real-Time Reverse Transcription Polymerase Chain Reaction (RT-PCR) Analysis of Clock Genes

Gene expression was analyzed using peripheral blood mononuclear cells (PBMCs). Blood samples were collected in ethylenediaminetetraacetic acid (EDTA) tubes. PBMCs were isolated by density gradient centrifugation at 3000 rpm for 30 min in Ficoll–Paque medium (GE Healthcare Life Sciences, Pittsburgh, PA, USA). Total RNA was extracted using the TRI reagent, and cDNA was synthesized with 1 μg of total RNA using an RT-PCR Kit (Takara Bio Inc., Shiga, Japan). PCR was performed using a Thermal Cycler Dice Real-Time System (Takara Bio Inc., Shiga, Japan). Amplification was performed under the following conditions: 40 cycles of denaturation at 95 °C for 10 s, annealing at 60 °C for 10 s, and extension at 72 °C for 10 s. Relative gene expression was analyzed using the comparative Ct method. Gene expression was determined using the 2^−^ΔΔCt method as the fold difference between ΔCt of the target sample and ΔCt of the calibrator sample. All reactions were performed in triplicates.

### 2.7. Statistical Analysis

Data are presented as mean ± standard deviation (SD) for continuous variables or number (percentage) for categorical variables. Differences in the baseline characteristics between the two groups were compared using an independent *t*-test. The Chi-squared test was used to compare differences in categorical variables, including smoking status, drinking status, exercise, diabetes, hypertension, and dyslipidemia, between the two groups. Differences within the groups after the two-week period were compared using the paired *t*-test. Differences in changes between groups were identified using linear regression to adjust for each baseline value. Restricted cubic spline analysis was used to determine the change patterns of sleep parameters during the two weeks. Two-sided *p*-values less than 0.05 were considered statistically significant. All statistical analyses were performed using SAS version 9.4 (SAS Institute Inc., Cary, NC, USA) and R software (version 4.1.1; R Foundation for Statistical Computing, Vienna, Austria).

## 3. Results

### 3.1. Patient Characteristics

Of the 14 participants in this study, 92.9% were women. Table 1 shows the baseline characteristics of the study population. Baseline characteristics were similar between control and LT groups in terms of age (37.0 ± 10.8 years vs. 47.4 ± 6.7 years, respectively; *p* = 0.054) and sex (6 women vs. 7 women; *p* > 0.999). Underlying diseases such as diabetes, hypertension, and dyslipidemia were similar between the two groups. Smoking, alcohol consumption, and physical activity habits were also similar between the two groups.

### 3.2. Changes in Biochemical and Physical Parameters

Table 2 shows the changes in the biochemical and heart rate variability parameters in the two groups before and after the two-week period after adjusting for each baseline value. A significant decrease in the fasting glucose level was noted in the control group (from 98.0 ± 5.3 mg/dL to 91.1 ± 8.1 mg/dL, *p* = 0.031). The adjusted mean changes in the fasting glucose level did not differ between the two groups after adjusting for each baseline value. Serum cortisol levels decreased in the LT group (9.7 ± 3.6 μg/dL vs. 7.6 ± 3.1 μg/dL, difference = −2.1 ± 2.7, *p* = 0.085), but the difference was not statistically significant. Moreover, the adjusted mean change in serum cortisol levels was greater in the experimental group than in the control group, although the difference was not statistically significant (mean changes in difference, −0.2 ± 2.2 μg/dL in control vs. −2.1 ± 2.7 μg/dL in LT group, *p* = 0.073).

### 3.3. Changes in Sleep Patterns and Quality

**Figure 2:**
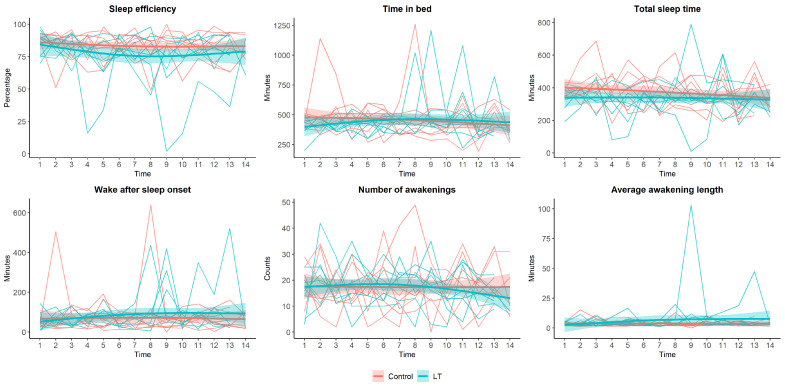
Sleep parameters assessed by actigraphy during the two weeks. Red line: control group, blue line: light therapy (LT) group.

Figure 2 shows the restrictive cubic spline curve of sleep parameters assessed by actigraphy over two weeks. There were no significant changes in sleep efficiency, time in bed, total sleep time, wake after sleep onset, number of awakenings, or average awakening length. Although there were no significant differences between the two groups, we found that the number of awakenings tended to decrease in the LT group.

**Figure 3:**
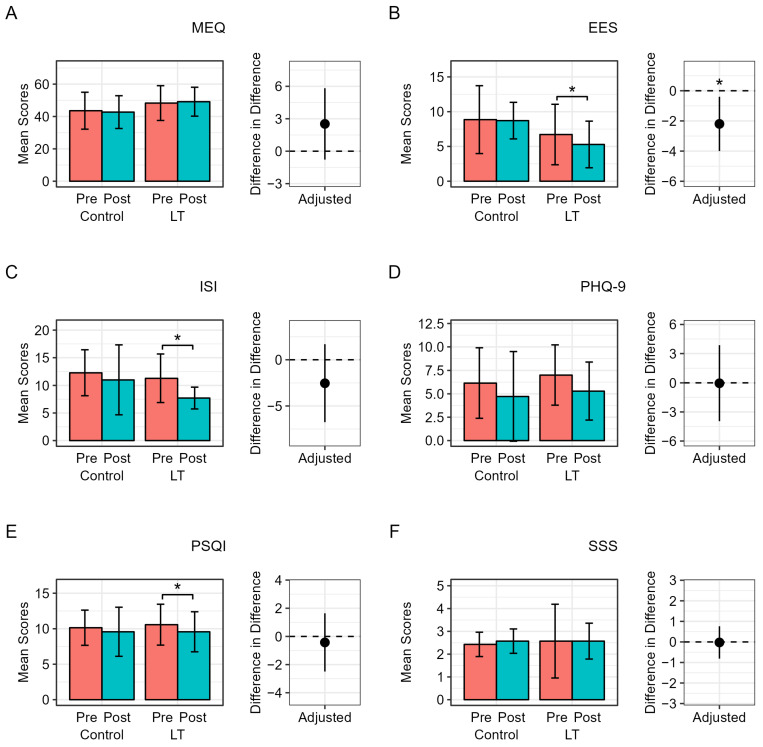
Changes in sleep− and mood−related parameters during the clinical trial. LT, light therapy. (**A**) Morningness–Eveningness Questionnaire (MEQ,), (**B**) Epworth Sleepiness Scale (ESS), (**C**) Insomnia Severity Index (ISI), (**D**) Patient Health Questionnaire (PHQ-9), (**E**) Pittsburgh Sleep Quality Index (PSQI), and (**F**) Stanford Sleepiness Scale (SSS) mean scores pre and post intervention in the experiment group (LT) and control group. Difference in difference between groups were calculated using linear regression to adjust for each baseline value. * denotes significant difference in the differences between two groups, * *p* < 0.05.

Figure 3 shows the changes in the metrics related to sleep and mood assessed using questionnaires in both groups after adjusting for each baseline score. In the control group, there were no significant changes in ESS, ISI, PSQI, SSS, MEQ, and PHQ-9 scores before and after the two-week period. In the LT group, the ESS, ISI, and PSQI scores significantly improved after the intervention (ESS: 6.7 ± 4.3 to 5.3 ± 3.4, *p* = 0.025; ISI: 11.3 ± 4.4 to 7.7 ± 2.0, *p* = 0.024; PSQI: 10.6 ± 2.9 to 9.6 ± 2.8, *p* = 0.004). There were no significant changes in the MEQ, PHQ-9, or SSS scores. However, when comparing the two groups, only the improvement in the ESS score was significantly higher in the LT group compared to the control group (mean difference, −0.14 ± 3.19 in control group vs. −1.43 ± 1.27 in LT group, *p* = 0.021) after adjusting for each baseline score.

### 3.4. Changes in Clock Genes’ Expression

**Figure 4:**
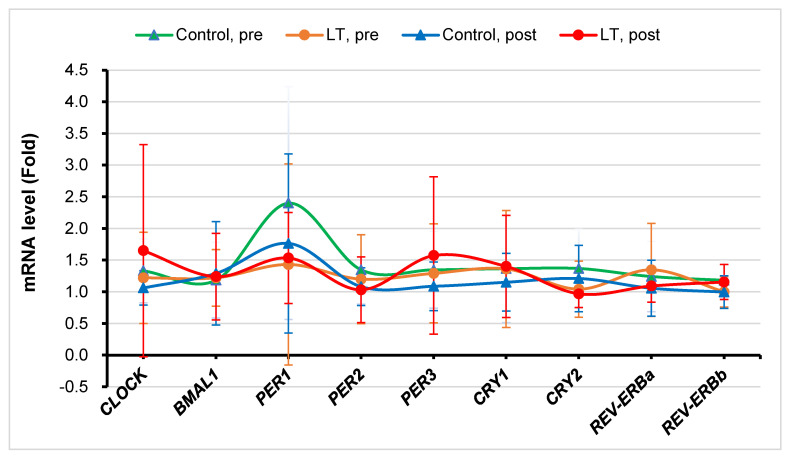
Clock genes’ expression in peripheral blood mononuclear cells before and after the two-week period. Triangles for control group and circles for the light therapy (LT) group. Control, pre: before the clinical trial in the control group; Control, post: after the clinical trial in the control group; LT, pre: before the clinical trial in the light therapy group; LT, post: after the clinical trial in the light therapy group.

Figure 4 presents the clock genes’ expression levels in PBMCs before and after the two-week period. Although there were no statistically significant changes in clock genes’ expression in both groups, the mean level of the CLOCK gene mRNA was decreased (1.33 to 1.06, difference = −0.269) in the control group, while it was increased (1.22 to 1.65, difference = 0.429) in the LT group. In a similar fashion, the mean level of the PER1 gene mRNA was decreased (2.40 to 1.76, difference = −0.637) in the control group, while it was increased (1.43 to 1.53, difference = 0.102) in the LT group. Furthermore, the mean level of the PER3 gene mRNA was decreased (1.35 to 1.09, difference = −0.260) in the control group, while it was increased (1.29 to 1.57, difference = 0.282) in the LT group.

### 3.5. Compliance and Safety

All the participants completed the study (*n* = 14). The safety assessment consisted of an evaluation of adverse events. Although one person reported mild glare, there were no serious adverse events.

## 4. Discussion

In this study, we investigated the effects of morning blue LT on sleep-related parameters and mechanisms in adults with insomnia. Light has a strong, non-visual impact on behavioral and physiological functions, including sleep–wake regulation, cognitive function, and hormone secretion. These light-dependent effects are mediated mainly by intrinsically photosensitive retinal ganglion cells (ipRGCs), which contain the photopigment melanopsin. The maximum sensitivity of ipRGC is in the short-wavelength blue region of the spectrum (approximately 460–480 nm), commonly referred to as “blue light”. Classical markers of the circadian system (cortisol and clock genes) as well as subjective (self-report) and objective (actigraphy) outcomes related to sleep were studied to determine the effectiveness of LT, specifically its effects on the human circadian system using monochromatic blue-light illumination. Some improvements in subjective and objective sleep-related parameters observed in the LT group in this study are consistent with previous findings [31,32], showing that exposure to bright blue-rich morning light improves daytime sleepiness and rapid alertness. This supports the potentially positive effects of LT on insomnia.

In the present study, we were unable to confirm the impact of LT on other objective sleep parameters such as sleep efficacy, total sleep time, and wake after sleep onset, as well as subjective parameters including PSQI, ISI, and SSS. The effectiveness of LT could be influenced by several factors, including circadian rhythm, light intensity, and light duration [21]. In addition, age, underlying diseases, medication, and individual variations in circadian rhythm sensitivity can influence the effectiveness of light therapy for insomnia.

These elements could contribute to the negative results of a limited effectiveness of LT for insomnia in the current study. While there were no significant differences in sleep efficiency, time in bed, total sleep time, wake after sleep onset, number of awakenings, or average awakening length, it is noteworthy that we did observe a tendency for a decrease in the number of awakenings in the LT group. This observation suggests a potential positive effect of LT on reducing sleep fragmentation, which merits further exploration in larger-scale studies with longer intervention periods.

Yeung et al. [33] also reported a placebo effect when assessing self-reported insomnia. This suggests that it may be more challenging to detect the exclusive effect of LT on insomnia or that a larger sample size may be required. In addition, light exposure not only during the morning but also at night should be considered.

We also evaluated the effect of LT by measuring serum cortisol levels. Cortisol levels in the LT group were lower than those in the control group, showing a potential trend for the effect of LT on cortisol. This is in line with previous studies showing that cortisol levels decreased after exposure to morning light [34]. However, the observed trend was not statistically significant, which may be due to the timing of light exposure being too late to elicit cortisol secretion and the small sample size. Gabel et al. [35] reported that the effect of blue light on cortisol is only effective when applied early in the morning, near the wake-up time. Sheer et al. [36] also suggested that the inconsistent results of cortisol changes are because cortisol levels are affected by not only the duration and light intensity but also the biological timing of LT exposure. Future studies should consider these factors to better elucidate the effects of morning blue LT on cortisol levels.

We also examined salivary melatonin levels in both groups on the first day and the last day of the clinical trial and found a tendency toward phase advancement of the melatonin circadian rhythm in the LT group in comparison to the control group. No significant changes were observed in either group. Most previous studies have focused on the effects of nighttime light exposure on non-visual-forming responses, in which light recognized by ipRGCs projects through the SCN in the brain and affects melatonin secretion in the pineal gland, which is very sensitive to short-wavelength light [37]. Further studies incorporating both morning and night light exposure are required.

To date, few studies have investigated the relationship between clock gene expression and LT. Rabia et al. [38]. found a decrease in PER1 and PER2 in patients with chronic insomnia and shift-working healthcare personnel, and they reported that the decrease in PER1 and PER2 gene expression is a result of short sleep periods. Based on previous research, in this study, we investigated a correlation between LT and clock genes’ expression. Overall, as a result of the *t*-test, we could not find significant differences in the expression levels of clock genes between the LT and control groups. In the case of the PER1 gene, the mean level of mRNA slightly increased from 1.43 to 1.53 in the LT group, whereas the mean level significantly decreased from 2.40 to 1.76 in the control group. This pattern may be interpreted as LT being effective. However, the PER2 gene showed a tendency to decrease in both the LT and control groups, and the level of decline was similar. In other words, a potential effect of LT was observed on the PER1 gene but not on the PER2 gene. Thus, to confirm the statistical significance of this tendency, additional studies are needed. Further studies on improving the protocol are needed to minimize the variability of clock genes between individuals [39,40] and to investigate the expression of various clock genes in the peripheral blood under various conditions, including the timing, duration, and intensity of light exposure.

This study had several limitations. First, because we conducted this exploratory study to investigate the effect of morning LT on sleep-related parameters and the circadian system, the sample size was small. However, in this exploratory study, we observed meaningful changes in daytime sleepiness. The power was 0.531 (for ESS). Based on the current study, we are attempting to design another clinical trial with a sufficient sample size. Second, this study only involved a short trial period of two weeks, and it is still unclear whether LT has long-term efficacy in the treatment of patients with sleep disorders.

Third, because light exposure is part of daily life, it is difficult to completely exclude the interference effect of artificial light. Although light is the most powerful zeitgeber, a stimulus synchronizing internal with external time, phase advancement can also occur during daytime physical exercise. Roenneberg et al. [41] reported, in a study using questionnaire data, that sleep time was increased by approximately 30 min when additional time was spent outdoors, but the relative contribution of light and physical activity to sleep has not yet been established. Fourth, it was difficult to exclude the influence of physical activity during the day during the study participation period because it was difficult to assess. It is known that blue light emitted from screens of digital devices can cause sleep disorders by reducing melatonin secretion [42]; however, the participants of this study were not subject to strict restrictions on daily life, such as suppression of the use of digital devices at night. Lastly, while we utilized self-reported MEQ questionnaires to assess circadian preference, it is important to incorporate objective measures of circadian rhythm. In the current study, we were unable to account for individual variations in circadian rhythms. We also did not evaluate the detailed typology of the patients’ sleep disorders. Thus, future research will require a study on personalized light exposure time, considering the circadian rhythm variations among individuals and the types of sleep disorders of the patients.

Despite these limitations, the present study is the first to comprehensively examine the influence of morning LT on sleep-related parameters, hormones, and circadian genes.

## 5. Conclusions

In this open-label feasibility study, we found that morning LT improved daytime sleepiness in adults with insomnia. We suggest that morning LT could improve the quality of life of adults with daytime sleepiness. Further large, randomized, controlled trials are needed to identify the impact of LT on the typology of sleep disorder, sleep parameters, hormones, circadian rhythm, and other individual variations.

## Figures and Tables

**Table 1:** Baseline characteristics of the study population.

Variable	Overall (*n* = 14)	Control Group (*n* = 7)	Experimental Group (*n* = 7)	*p*-Value
Age, years	42.2 ± 10.2	37.0 ± 10.8	47.4 ± 6.7	0.054
Gender, *n* (%)				>0.999
Women	13 (92.9)	6 (85.7)	7 (100.0)	
Men	1 (7.1)	1 (14.3)	0 (0.0)	
Weight, kg	62.1 ± 12.2	60.0 ± 13.1	64.3 ± 11.7	0.533
Height, m	161.9 ± 8.7	162.4 ± 10.5	161.5 ± 7.4	0.863
Body mass index, kg/m^2^	23.5 ± 3.1	22.5 ± 2.2	24.6 ± 3.7	0.217
SBP, mmHg	121.9 ± 9.4	119.4 ± 6.7	124.3 ± 11.6	0.360
DBP, mmHg	74.3 ± 7.1	71.1 ± 7.2	77.4 ± 5.9	0.102
Diabetes, *n* (%)				>0.999
Yes	1 (7.1)	0 (0.0)	1 (14.3)	
No	13 (92.9)	7 (100.0)	6 (85.7)	
Hypertension, *n* (%)				
Yes	0 (0.0)	0 (0.0)	0 (0.0)	
No	14 (100.0)	7 (100.0)	7 (100.0)	
Dyslipidemia, *n* (%)				0.445
Yes	2 (14.3)	0 (0.0)	2 (28.6)	
No	12 (85.7)	7 (100.0)	5 (71.4)	
Smoking, *n* (%)				>0.999
Yes	1 (7.1)	1 (14.3)	0 (0.0)	
No	13 (92.9)	6 (85.7)	7 (100.0)	
Drinking, *n* (%)				>0.999
Yes	9 (64.3)	4 (57.1)	5 (71.4)	
No	5 (35.7)	3 (42.9)	2 (28.6)	
Exercise, *n* (%)				>0.999
Yes	9 (64.3)	4 (57.1)	5 (71.4)	
No	5 (35.7)	3 (42.9)	2 (28.6)	

Experimental group, LT. *p*-value was calculated using the independent two-sample *t*-test.

**Table 2:** Characteristics and changes in the control and experimental groups (LT) at baseline and after the intervention.

Variable	Control Group				Experimental Group (LT)				*p*-Value †
	Pre	Post	Diff	*p*-Value *	Pre	Post	Diff	*p*-Value *	
WBC	6.8 ± 2.4	6.0 ± 1.1	−0.8 ± 1.8	0.284	7.1 ± 1.7	7.1 ± 1.4	−0.1 ± 0.6	0.810	0.059
Glucose, mg/dL	98.0 ± 5.3	91.1 ± 8.1	−6.9 ± 6.5	0.031	109.0 ± 15.0	101.6 ± 10.4	−7.4 ± 8.6	0.062	0.369
TC, mg/dL	170.1 ± 27.5	168.3 ± 16.0	−1.9 ± 23.3	0.840	186.3 ± 30.9	185.9 ± 29.5	−0.4 ± 4.0	0.784	0.404
TG, mg/dL	188.4 ± 252.9	103.1 ± 41.2	−85.3 ± 227.9	0.360	168.3 ± 133.4	120.0 ± 31.9	−48.3 ± 131.5	0.369	0.313
HDL-C, mg/dL	57.6 ± 10.5	61.4 ± 8.3	3.0 ± 9.2	0.308	65.3 ± 10.7	64.7 ± 12.7	−0.6 ± 7.5	0.847	0.658
LDL-C, mg/dL	94.7 ± 24.8	99.7 ± 15.5	5.0 ± 17.8	0.484	109.9 ± 34.3	113.0 ± 31.5	3.1 ± 11.7	0.505	0.743
Insulin, IU/L	16.2 ± 16.6	12.7 ± 11.0	−3.5 ± 6.8	0.225	14.9 ± 11.2	7.2 ± 3.5	−7.7 ± 11.9	0.138	0.132
HOMA IR	4.0 ± 4.0	2.8 ± 2.3	−1.6 ± 2.1	0.198	4.2 ± 3.4	1.8 ± 1.0	−2.4 ± 3.5	0.120	0.187
HsCRP, mg/dL	0.6 ± 0.5	0.5 ± 0.5	−0.1 ± 0.5	0.700	0.8 ± 1.5	1.1 ± 1.7	0.4 ± 1.6	0.577	0.409
Cortisol, μg/dL	11.0 ± 4.5	10.8± 3.7	−0.2 ± 2.2	0.792	9.7± 3.6	7.6 ± 3.1	−2.1 ± 2.7	0.085	0.073
ACTH	24.2 ± 17.4	22.0 ± 10.5	−2.2 ± 9.7	0.575	27.5 ± 14.4	24.5 ± 9.7	−3.0 ± 12.0	0.526	0.818
Serotonin	165.0 ± 73.5	168.2 ± 97.9	3.2 ± 71.6	0.909	136.6 ± 132.5	146.0 ± 115.0	9.4 ± 39.6	0.551	0.950
MHR (bpm)	86.9 ± 16.1	89.6 ± 8.4	2.6 ± 8.4	0.436	75.4 ± 7.8	77.3 ± 8.8	1.9 ± 6.9	0.488	0.089
SDNN (ms)	27.1 ± 16.6	25.3 ± 7.5	−1.8 ± 14.0	0.749	29.0 ± 11.4	28.4 ± 13.0	−0.6 ± 5.2	0.762	0.639
TP (m/s^2^)	761.7 ± 842.5	438.6 ± 275.6	−323.0 ± 830.5	0.343	805.1 ± 634.4	870.9 ± 984.9	65.7 ± 685.1	0.808	0.273
LFNorm	60.6 ± 16.9	36.8 ± 11.7	−23.8 ± 16.8	0.009	56.1 ± 27.7	51.1 ± 19.6	−5.1 ± 30.5	0.674	0.115
HFNorm	39.4 ± 16.9	63.2 ± 11.7	23.8 ± 16.8	0.009	43.9 ± 27.7	49.0 ± 19.6	5.1 ± 30.5	0.674	0.115
LF/HF Ratio	2.4 ± 2.5	0.6± 0.4	−1.7 ± 2.5	0.118	2.7 ± 3.3	1.5 ± 1.3	−1.2 ± 3.6	0.402	0.147

WBC, white blood cells; TC, total cholesterol; TG, triglyceride; HDL-C, high-density lipoprotein cholesterol; LDL-C, low-density lipoprotein cholesterol; HOMA-IR, homeostatic model assessment for insulin resistance; hsCRP, high sensitive C-reactive protein; ACTH, adrenocorticotropic hormone; MHR, mean heart rate; SDNN, standard deviation of normal-to-normal interval; TP, total power; LFnormal, normalized low frequency; HFnormal, normalized high frequency. * *p*-value was calculated using the paired *t*-test. † *p*-value for difference in the differences between two groups.

## Data Availability

The datasets generated and analyzed during the current study are available from the corresponding author upon reasonable request.

## Supplementary Materials

The following supporting information can be downloaded at: https://www.mdpi.com/article/10.3390/medicina59061066/s1, Figure S1: Photometer assessment results by the Korean Photonics Technology Institute.
