# Supplementary material for: Feasibility and Efficacy of Morning Light Therapy for Adults with Insomnia: A Pilot, Randomized, Open-Label, Two-Arm Study
Source: Medicina (Kaunas). 2023 Jun 1;59(6):1066. doi: 10.3390/medicina59061066 (PMC10305051; doi:10.3390/medicina59061066)
Supplement: Supplementary file 1 [file medicina-59-01066-s001.zip › medicina-2365840-supplementary.docx]

Supplementary materials

**
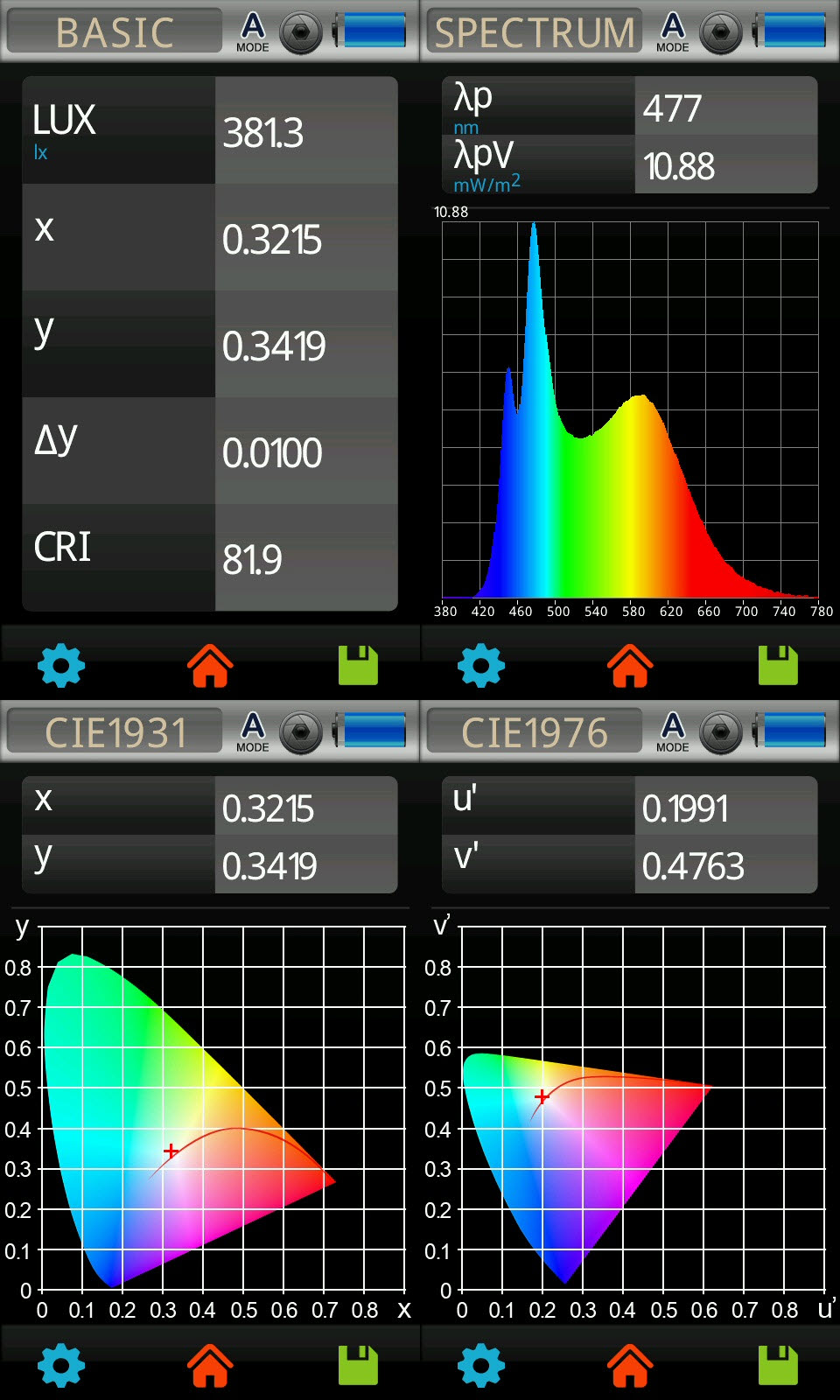
.**

**Figure S1.** Photometer assessment results by the Korea Photonics Technology Institute.
